# Community Interventions to Improve Cooking Skills and Their Effects on Confidence and Eating Behaviour

**DOI:** 10.1007/s13668-016-0185-3

**Published:** 2016-10-17

**Authors:** Ada L. Garcia, Rebecca Reardon, Matthew McDonald, Elisa J. Vargas-Garcia

**Affiliations:** 1 Human Nutrition, School of Medicine, Dentistry and Nursing, College of Medical, Veterinary and Life Sciences, University of Glasgow, Room 3.06, Level 3, New Lister Building, Glasgow Royal Infirmary, 10-16 Alexandra Parade, G31 2ER Glasgow, UK; 2 Eden Care Centre, Carecorp Seniors ServicesVancouver Canada, 01-1525 Robson Street, Vancouver, BC V6G 2C3 Canada; 3 Nutritional Epidemiology Group, School of Food Science and Nutrition, University of Leeds, School of Food Science & Nutrition, LS2 9JT Leeds, UK

**Keywords:** Cooking programmes, Evaluation, Confidence, Fruit and vegetable, Healthy eating

## Abstract

**Purpose of Review:**

Community-based interventions aiming to improve cooking skills are a popular strategy to promote healthy eating. We reviewed current evidence on the effectiveness of these interventions on different confidence aspects and fruit and vegetable intake.

**Recent Findings:**

Evaluation of cooking programmes consistently report increased confidence in cooking skills in adults across different age groups and settings. The effectiveness of these programmes on modifying eating behaviour is less consistent, but small increases in self-reported consumption of fruit and vegetables are also described. Lack of large samples, randomization and control groups and long-term evaluation are methodological limitations of the evidence reviewed.

**Summary:**

Cooking skill interventions can have a positive effect on food literacy, particularly in improving confidence on cooking and fruit and vegetable consumption, with vulnerable, low-socieconomic groups gaining more benefits. Consistency across study designs, delivery and evaluation of outcomes both at short and long terms are warranted to draw clearer conclusions on how cooking programmes are contributing to improve diet and health.

## Introduction

Poor diet is a major risk factor linked to obesity and other comorbidities. Low education attainment, low income and high socioeconomic deprivation are main factors associated with poor diet [1, 2]. These factors tend to increase the likelihood of inadequate food access, low food and nutrition literacy and lack of practical cooking skills in economically deprived households [3]. Currently, there is growing evidence linking home cooking with healthier dietary choices, particularly for higher intakes of fruits, vegetables, and whole grains [4]; whilst eating outside the home has been associated with an increased consumption of ready-to-eat meals and calorie-dense convenient foods [5]. Considering that ‘not knowing how to cook’ stands as barrier to healthful food preparation [6], the delivery of community cooking skill programmes has gained attention in public health agendas as a vehicle to improve and promote confidence, well-being, and enhance meal quality and preparation practices [7••, 8]. These programmes have increased and continue to increase in popularity because they offer a valuable channel to engage with vulnerable groups via inclusive social activities, whilst positively impacting their dietary profiles and health outcomes [8].

The term ‘cooking skills’, within public health nutrition, has been generally used to portray a combination of mechanistic and physical skills that are applied during home food preparation, such as ‘chopping vegetables’, ‘stir-frying’, or ‘cooking rice’ [9]. Nevertheless, the term has also been recognised to encompass the accomplishments beyond technical activities, including preparation, conceptual and perceptual abilities on food handling, safety and storage, and other factors related to chemistry and nutrition [9, 10]. Likewise, the emerging concept of ‘cooking competence’, as a shift from the traditional technical-centred approach, has been proposed as a merge between knowledge and skills to enable nutritious meal preparations whilst concomitantly incorporating aspects of planning, budgeting, storing, eating, and waste disposing [10]. Condransky and Hegler refer to the former as ‘culinary nutrition’ [11] and have further emphasised that pairing nutritional knowledge with practical demonstrations is warranted to achieve changes in eating behaviours, as the provision of information alone has been found to be ineffective to bring about behavioural change [12]. Recently, the term ‘food literacy’ has been proposed as a concept that covers all definitions described above: cooking skills, cooking competences, and culinary nutrition. Indeed ‘food literacy’ encompasses a more holistic approach to describe the practicalities needed to meet nutrition recommendations: plan, management, selection, preparation, and consumption.

The emerging interest on cooking skills has coincide with a concern in the marked declines in home cooking as reported in the UK in the 1980s [13]. These reductions were also observed in the in the UK [14] and the USA [15] after curricular culinary lessons were removed from educational platforms. In Canada, the re-introduction and reinforcement of cooking skill programmes using the ‘school as a community’ approach showed effective on engaging children, adolescents, and parents in food literacy and meal preparation whilst strengthening local ownership [16]. Additionally, a number of national and local campaigns have been established in several Western countries to promote cooking skill strategies amongst vulnerable, low-income communities such as, ‘What’s Cooking’ [17], ‘Get cooking’ [18], ‘Cooking Matters’ [19], ‘Jamie’s Ministry of Food’ [20], and the ‘Stephanie Alexander Kitchen Garden (SAKG) Program’ [21].

Whilst the evaluation of public health interventions is necessary to identify their effectiveness, accountability, and adaptation, the evidence on the short- and long-term impacts and sustainability of community cooking skill programmes remains limited [22••]. Evidence suggests large heterogeneity in the structure and delivery of cooking programmes, and this varies in target populations, settings, course content and length, modes of delivery, and the outcomes measured; nevertheless, most of these initiatives include outcome elements related to confidence, skills, and eating behaviours. The present narrative review provides an overview on the effectiveness of community cooking skill interventions on cooking confidence and eating behaviours namely fruit and vegetable intake, which is often used as a proxy for a healthy diet.

### Main Outcomes of Cooking Skill Programmes: Eating Behaviours

Interventions have been heterogeneous in the activities delivered to meet groups’ needs, which is inherent to their own planning and funding [22••]. However, as portrayed in Tables 1 and 2, most of the initiatives have been conducted in the UK, Australia, and USA, with adults often being a secondary target to reach children. Indeed, there is emphasis placed over parental involvement on delivery of school-based interventions, as they are perceived as a key feature to impacting children’s eating behaviours and confidence in home cooking [31]. Alternatively, approaches, like in the Jamie’s Ministry of Food [20] initiative, which focus on teaching young adults basic food preparation skills and nutritional knowledge to influence healthfulness of meal preparation within the family home, can be modelled and transferred to others. Yet, debate still exists [13] over which targeted populations—children, adolescents, adults, or seniors—would most require and benefit from these interventions and so as to tailor programme development (gaining a ‘best fit’) and resource allocation (a ‘best buy’).

**Table 1:** Summary of evidence of cooking skill effectiveness on eating behaviours: quasi-experimental study designs

Study	Country and study design	Number of participants intervention/control		Age	Aims	Intervention content	Duration	Measurement method	Outcomes	Completers*
Flego et al. 2014 [7••] Herbert et al. 2014 [23]	Ipswich, Australia quasi-experimental	1526	434	46–48 years	Evaluate impact of JMoF cooking skill programme on food preparation and healthy eating practices, beliefs, confidence, knowledge and behaviours	90-min sessions delivered to low SES, likely overweighed adults focused on preparing basic meals that are easy to cook, fresh and healthy. control waiting list	Intervention 10 weeks follow-up 6 months	Semi-structured interviews, self-reporting questionnaires	At post-intervention intervention vs control group reported improvements in cooking confidence (*p* < 0.001) as well as increase in vegetable consumption by 0.5 portions/day; and serving and frequency of cooking meals from scratch (*p* = 0.001). sustained outcomes at follow-up in intervention group	53 %
Wrieden et al. 2007 [3]	Scotland, UK quasi-experimental	51 at post-intervention 36 at follow-up	42 at post-intervention 27 at follow-up	32	Evaluate impact of Cook Well programme on cooking confidence, food preparation and choice	120- min sessions delivered to low SES adults focused on food handling, nutrition, tasting and healthy meal preparation control waiting list	Intervention 7 weeks follow-up 6 months	7-day diary self-administered questionnaires	Intervention vs control group exhibited: marginal increase in fruit intake by 1 portion/week (*p* = 0.047) at post-intervention only and increased confidence following a recipe at follow-up.	68 %
Condrasky et al., 2006 [24]	South Carolina, USA quasi-experimental	15	14	25	Evaluate impact of ‘Cooking with a Chef’ programme on healthy eating through instructive lessons on nutrition, food selection	120-min sessions delivered to low SES parents/caregivers of pre-schoolers on culinary skills, taste tests, and practical cooking sessions. controls received printed materials (i.e. recipes)	Intervention 6 weeks	24-h food recall form food behaviour checklist programme evaluation form	Intervention vs control group exhibited: increased awareness/confidence on basic food preparation children increased number of fruit portions per day no significant changes in fruit and vegetable intake in parents from either group	70 %

*JMoF* Jamie’s Ministry of Food, *SES* socioeconomic status, *IG* intervention group, *CI* confidence interval, *CK* community kitchen

*% of participants providing answers at last measuring point

**Table 2:** Summary of evidence of cooking skill effectiveness on eating behaviours: single group pre- and post-test study designs

Study	Country and study design	Number of participants	Age	Aims	Intervention content	Duration	Measurement method	Outcomes	Completers*
Hutchinson et al. 2016 [25]	Leeds, UK single group pre-test/post-test	795 at post-intervention 462 follow-up	20–65 years	Evaluate impact of JMoF cooking skills programme on cooking and healthy eating practices, beliefs, confidence, knowledge and behaviours	90-min sessions delivered to young adults focused on concepts of healthy eating and meal preparation. payment to attend sessions £4.50–£7.50	Intervention 8 weeks follow-up 6 months	Self-administered questionnaires	At follow-up: significantly increases on F&V intake by 1.5 portions/day cooking confidence score increased by 1.7 (scale from 0–5) others: decreased consumption of snacks by less than one item a day.	58 %
Moreau et al. 2015 [26]	Quebec, Canada retrospective single group pre-test/post-test	144	>50	To evaluate if cooking workshops with nutritional education helped to improve dietary habits and confidence, knowledge and autonomy.	Eight weekly 2-h cooking sessions delivered which focused on providing appropriate nutritional advice to older adults. payment of $20 to attend all sessions	Intervention 8 weeks	Self-administered questionnaire	At post-intervention: small significant (*p* < 0.05) increases in nutritional knowledge (87 to 89 %), confidence to eat healthily (77.5 to 80.5 %) and eating behaviours (82 to 85 %) compared to baseline. this included an increased proportion of people consuming 5 or more fruit and vegetables per day (41 to 57 %, *p* < 0.05)	N/A 85.5 % attended 7 or more
Garcia et al. 2014 [27•]	Arran & Ayrshire, UK single group pre-test/post-test	102 at post-intervention 44 follow-up	17 and above	Evaluate impact of NHS community programme on cookery skills, eating behaviours, confidence and self-esteem.	120-min sessions delivered to nursery parents focused on healthy food preparation	Intervention 4–8 weeks follow-up: 12 months	Self-administered questionnaire telephone-based interviews	At follow-up: increased cooking confidence in using basic ingredients (med: 7v6, *p* = 0.033), following simple recipes (med: 7v7, *p* = 0.06) and cooking new foods (med: 6v6, *p* = 0.08) increased intake of F &V to ‘once a day’.	43 %
Abbot et al. 2012 [28]	Western Sydney, Australia single group pre-test/post-test	23	19–72	Evaluate a diabetes cooking course on food preparation and nutritional knowledge	240-min sessions delivered to Aboriginal females with (pre) diabetes focused on healthful eating preparation and provision of nutritional knowledge	Intervention 18 weeks	Semi-structured in depth interviews	At post-intervention: increases in food knowledge, cooking techniques (i.e. reductions in oil/fat use) and improvements in food literacy.	N/A
Keller et al. 2004 [29]	Ontario, Canada	19	>65	Evaluate impact of the ‘Men can cook!’ programme on cooking confidence, enjoyment, nutritional knowledge and food preparation	Monthly 120-min sessions delivered to community-living senior males on food literacy (i.e. meal planning, following a recipe, measuring ingredients) and nutritional knowledge.	Intervention 8 months	Semi-structured interviews, self-administered short questionnaire	At post-intervention,: based on a 5 point Likert scale small non-significant increases in cooking confidence (pre 2.6 vs post 2.8), trying new foods (3.9 vs 4)	
Beets et al., 2007 [30]	Oregon, USA single group pre-test/post-test	20	Not reported: ‘young adolescents’	Evaluate culinary camp initiative on development of culinary skills	240-min sessions delivered to adolescents to increase food preparation and knowledge	Intervention 8 days no follow-up	Self-administered questionnaire	Food preparation frequency remained unaltered, whereas significant improvements were reported in nutritional knowledge and perceived cooking ability (*p* = 0.04)	85 %

*JMoF* Jamie’s Ministry of Food, *SES* socioeconomic status, *F&V* fruit and vegetable, *IG* intervention group

*% of participants providing answers at last measuring point

The duration of cooking courses has varied from a week to 2 years, with all of them being group-based, which emphasises the social relevance of this mode of delivery to enhance stronger support networks, building capacity and feelings of cohesion and efficacy [32]. Alongside the variability of intervention components, the tools used to measure the progress on food-related outcomes and dietary practices are diverse. Most tools used, albeit validated, are comprised of self-administered questionnaires on dietary behaviours, completed by the participants or child’s parents; but some studies have used mixed methods to further explore the participants’ experiences of the programmes [25, 28, 33]. Some items captured included perceived changes in confidence to applying different cooking techniques, following a recipe, making a meal from raw ingredients (widely referred to as ‘from scratch’), the willingness to try new foods, changes in self-esteem, and questions on dietary practices reflected in usual intakes of snacks, take-away meals, and fruits and vegetables. All of the former reported on likert/agreement scales varying in point scores, which accentuate the lack of consistency in the development and evaluation of cooking skill-based health initiatives [34].

### Effects on Fruit and Vegetable Consumption

Increasing fruit and vegetable consumption is a key strategy to improve diet, yet intakes remain significantly below recommended levels, especially across socially disadvantaged groups who constitute a majority of the targeted populations for cookery initiatives [3, 9]. Cooking skill programmes have aimed to expose participants to new foods, as a means to increase variety and facilitate adherence to current dietary guidelines for fruit and vegetable consumption. In a study evaluating the impact of a 4–8-week cooking skill programme in a mainly rural adult population in Ayrshire and Arran, Scotland, sustained improvements in fruit and vegetable intakes were documented [27•]. Similarly, recent evidence has highlighted significant self-reported increases in daily fruit and vegetable intakes by 1.5 portions in adults after attendance to an 8-week cooking course in urban Leeds, England [25]. Nevertheless, the lack of randomisation to a comparator or control group in both studies does not allow causality between exposure to the cooking sessions and outcomes to be established, as confounding is likely. Participants who attend cooking classes might be more motivated to change their behaviours, and reporting bias is a limitation in most cooking skill interventions, due to the self-reported nature of the questionnaires administered [22••, 35•]. Additionally, individuals from lower socioeconomic groups might have a lower baseline intake and, thus, are prone to exhibit greater benefits/changes; however, this can also be considered a strength because these are the desired target groups.

A commonly shared feature across most cooking interventions is the opportunity to taste the foods produced at the end of each session. This strategy has shown promising outcomes for social bonding, linkage and encouragement of group discussion, whilst also offering a starting point for the modification of neophobic responses towards disliked, rejected or foods not eaten [36] such as fruit and vegetables. Exposure itself, or watching peers eating certain foods, provides participants with a modelling experience in which the behaviour can be enacted or copied. Qualitative results from the evaluation of the SAKG school-based programme highlighted an increase in children’s willingness to try new foods with exposure strategies, particularly vegetables [21]. Nevertheless, in the SAKG programme, parental reports highlighted only small improvements in consumption patterns, with 70 % of the children still not having at least two daily servings of fruit and less than 10 % meeting the Australian recommendation of five portions of vegetables per day. The ‘Edible School Garden’ at the Berkeley School district in the USA offers another example which integrates gardening activities together with a food preparation component [15]. This programme, founded by celebrity chef Alice Waters, seeks the exposure of children to growing, cooking and tasting new foods whilst slowly integrating these activities into the regular school curriculum. Three-year follow-up results revealed that children at schools with higher involvement in the initiative (offering up to 1.5 h per week for cooking and gardening instruction vs no practical cooking sessions) had increased fruit and vegetable intake by more than one serving daily in comparison to students with lesser developed school activities. However, as effects weakened during the transition from elementary to middle school, maintenance of this initiative was emphasised as part of the programme enhancements through pre-adolescence. Furthermore, evidence from two systematic reviews [35•, 37] has also indicated the short-term success of cooking initiatives to increase participants’ range of preferences and self-reported intakes of fruit and vegetables after repeated exposures.

### Cooking Confidence: What Is It and How Is It Measured?

One of the main aims of cookery programmes has been to increase participants’ cooking confidence. The concept varies in meaning as it may involve the ability to adequately measure ingredients, cut up fruits and vegetables, follow a recipe, use fresh ingredients or be comfortable with basic culinary techniques [38].

In two studies evaluating the effectiveness of Jamie’s Ministry of Food 10-week community-based cooking skill programme in Ipswich, Australia [7••, 23], results showed an increase in cooking confidence, psychosocial factors, food procurement behaviour, healthier cooking and enjoyment in meal preparation at the end of the programme and 6-month follow-up. Evaluation of the same initiative in the UK also highlighted increases in confidence scores for both assessment periods [25]. However, the latter study may have had a skewed sample with participants having previous cooking experience, as expressed by certain comments on advanced culinary sessions desired and considered that a fee was required to be enrolled in the course.

Work from Laska and colleagues [39] on a cohort of 1321 individuals living in urban Minnesotta, USA followed from youth until adulthood also highlighted that a higher frequency of food preparation during adolescence was associated with increased likelihood of enjoying cooking in their mid-late twenties (β=0.18, *p* < 0.01). Furthermore, adolescents that assisted with dinner preparation at least once or twice per week, in comparison to those that did not, were significantly more likely to buy fresh vegetables (19.4–33.9 %, *p* < 0.001) and prepare a meal with chicken, fish or vegetables (44.9–52.4 %, *p* = 0.01) in emerging adulthood. Though some behaviours did not appear to track in later years, it is suggestive that exposure to cooking in teenage years could influence confidence and other health behaviours in subsequent years to some extent.

Evaluation of a single-arm cooking intervention in male seniors from a retirement centre in Canada [29] indicated modest improvements at follow-up (8 months after commencement of the programme) in attitude towards healthful meal preparations, increased confidence in cooking more complex dishes and decreased food neophobia. Whilst changes from baseline to follow-up remained non-significant, interviews with participants emphasised that the majority gradually had developed multiple skills and healthier culinary strategies. The authors attributed a failure to detect significant differences after the programme to the small sample size (*n* = 19) and difficulties to bring about change in men’s perception of their own culinary abilities. It is also possible that these results were influenced by participants’ initial description of their own skills, with 70 % of them indicating knowing how to prepare basic dishes. A further study in older adults also in Canada used a larger sample (*n* = 144) and showed small but significant increases from baseline to post-intervention in nutritional knowledge, confidence to eat healthy and eating behaviours, including a higher number of participants achieving five or more fruit and vegetables a day [26]. Interestingly, the reported baseline values were already high and a high completion rate was reported. This could have been due to a monetary incentive offered for completion. Nevertheless, the reviewed studies aimed at senior participants show positive outcomes in both confidence and eating behaviours.

Previous systematic reviews [22••, 31, 35•, 37, 38] have shown short-term improvements in cooking confidence both in children and adults taking part in culinary interventions. Nevertheless, measurement of confidence remains problematical as self-perceived level of confidence by participants may not coincide with their actual skill level, and so evaluation of skills parallel to attitudes has been highlighted as a more objective indicator of programme outcomes [31].

In 2015, Community Food and Health Scotland (CFHS) published a report documenting a comprehensive review of the grey literature, using a realist synthesis approach, to seek out which strategies/approaches aided the most in achieving the outcomes/goals of cooking skill programmes targeting vulnerable and low-income populations. The results identified course practitioners as key elements in delivering evidence-based practice to strengthen, target, tailor and reinforce programmes via multiple and diverse strategies in a variety of settings. These included, but not limited to, encouraging participants to influence content/recipe selection, adjusting session focuses, having tasting periods, eating together at the end of class and supporting peers in the learning process through social interactions. They also found that the theories of outcome expectance, personal relevance, positive attitudes, self-efficacy and descriptive and subjective norms were used more regularly to strengthen and focus behavioural changes. Whilst positive, the review did uncover that most of the literature lacked clarity in the plans, implementation and evaluation (methods and tools) of the cooking skills programmes, which in turn can impede outcome measure conclusions, duplicability and ultimately further funding of the programme. They concluded that cooking activities delivered by community-based initiatives were successful on targeting and reaching low-income and vulnerable groups and that good practice by deliveries was consistent, and some of them used specific concepts related to behaviour change theory, ie. self-efficacy, salience and social norms were used more frequently than goal setting [19].

The introduction of new technologies (such as those integrating image-based methods including videos or photos) could possibly provide another approach to measure outcomes of cooking interventions; yet, considering the vulnerability of the targeted groups, the use of such methods (often more invasive) could be detrimental to follow-up rates, as keeping ‘captive audiences’ remains one of the main challenges across most studies.

## Implications for Future Research

There is a plethora of public health nutrition interventions using the delivery of cooking skills as a practical element of nutrition promotion, which shows positive results on food literacy. However, the quality of the studies published until now reflects the complexity of performing studies involving free living individuals. The lack of interventions with rigorous study designs that use randomization or have control groups is an emerging problem faced by health practitioners who wish to improve their practice based on evidence. However, this needs to be considered in the context of complex interventions in free living subjects in which a rigorous RCT design proves impossible, thus finding alternative ways for generating evidence is guaranteed. Furthermore, evaluation plans are often not incorporated within programme delivery, resulting in a lack of data on process and longer term outcomes to support their sustainability [32]. Most evaluation tools used across studies have not been validated, are subjected to selection bias, are highly reliant on self-reporting or use varying definitions and measurements of eating/cooking behaviours [34]. These factors can make drawing conclusions on the effectiveness of cooking skill interventions to improve food preparation and health behaviours difficult [8].

Process evaluations are important to undertake to ensure that programmes are being implemented as planned, yet the assessment of cooking programmes competences is infrequently reported in the literature [8]. A study examining participatory experience, appeal, effectiveness and the operations of a cooking course 6 months to 5 years post-intervention in Aboriginal Australians with diabetes revealed that some areas of the intervention design may have reduced the course’s appeal, impacted on the objectives negatively, and were considered too structured for the population of interest [28].

Currently, the availability of long-term impact and process evaluation studies of cooking skill programmes is limited, and the needed for more is essential in order to help improve the success of present programmes, help better develop new programmes and sustain funding. Although randomised controlled trials are considered as the ‘gold standard’, the use of quasi-experimental designs inclusive of a comparator group may be more achievable whilst still enhancing statistical rigour, particularly when targeting hard-to-reach groups [40]. This approach could help clarify the potential for cooking skills to affect dietary behaviours, lead to healthy weight achievements and other health outcomes. Research projects could additionally benefit from the development of a theory of change in the context of evaluation of complex interventions. This means to develop an a-priory understanding of how and why a programme operates via involvement of all stakeholders, in particular the target population, should be at the core of this process. A further key feature is a thorough development and implementation of process evaluation. A theory of change will aid understand and refine the links across activities, assumptions in place and indicators of change as the intervention is developed [41].

Population targets for cooking skill interventions are often living in areas of social deprivation; but skill development alone will not reverse affordability constraints nor directly achieve behaviour change. However, these interventions do stand as a potential vehicle to improve dietary quality, particularly if used with nutrition promotion and incorporating behaviour change techniques. Indeed, whilst heterogeneous and small effects have been noted for increases in fruits and vegetables and other proxies of healthful eating, budgeting skills have been raised as a positive outcome following the programmes [8], despite existing financial barriers not being removed entirely.

## Conclusions

Our review suggests that brief cooking programmes are modestly effective on increasing confidence to perform skills that improve some aspects of food literacy, in particular those relevant to cooking meals (preparation). Interventions appear to be providing more benefits to vulnerable, low-income and socially deprived adults and their families, often one of the main target groups of cooking initiatives. Curricular food preparation sessions can positively expose and influence children to try new foods. However, variation in the content, definition and assessment of eating and cooking behaviours across studies limits current understanding of their likely efficacy and sustainability to impact other short-term dietary outcomes, including changes in fruit and vegetable intake.
